# Incorporating fine‐scale behaviors into habitat suitability modeling: A case study for sea turtles

**DOI:** 10.1002/eap.70095

**Published:** 2025-09-12

**Authors:** Jenna L. Hounslow, Sabrina Fossette, Arnold van Rooijen, Anton D. Tucker, Scott D. Whiting, Adrian C. Gleiss

**Affiliations:** ^1^ Centre for Sustainable Aquatic Ecosystems Harry Butler Institute, Murdoch University Murdoch Western Australia Australia; ^2^ Environmental and Conservation Sciences Murdoch University Murdoch Western Australia Australia; ^3^ Biodiversity and Conservation Science, Department of Biodiversity, Conservation and Attractions Kensington Western Australia Australia; ^4^ UWA Oceans Institute, University of Western Australia Perth Western Australia Australia; ^5^ School of Earth and Oceans, University of Western Australia Perth Western Australia Australia

**Keywords:** animal‐borne video, biologging, conservation planning, flatback turtle, macrotidal embayment, marine turtle, spatial management, supervised machine learning

## Abstract

Habitat suitability models (HSMs) are popular statistical tools used to inform decision‐making for conservation planning, using species location data to characterize species–environment relationships and identify important habitats. Suitable habitats may vary according to behavior‐specific resource requirements (e.g., foraging, resting), yet HSMs generally ignore behavior because obtaining spatially explicit behavioral data from wild animals is challenging. As such, suitable habitats may be incorrectly identified, and processes determining habitat selection may be misinterpreted. Despite offering unprecedented behavioral insight, contemporary multi‐sensor biologgers remain underutilized in this context. We incorporated behavior into HSMs using biologging data collected from adult flatback turtles *Natator depressus* (*n* = 42) at a macrotidal study site in Western Australia and subsequently identified and characterized suitable habitat for key in‐water behaviors. Foraging and resting locations derived from high‐resolution motion sensor data (e.g., accelerometer, magnetometer) coupled with animal‐borne video and GPS data were combined with 10 environmental features (i.e., bathymetry, aspect, slope, terrain ruggedness, distance from the coast and currents from a bespoke hydrodynamic model of the study site). A series of random forest HSMs were implemented for each behavior, accounting for temporal variation in habitat use. Bathymetry, distance from the coast, and currents best determined both foraging and resting suitability, with observed differences in habitat selection between behaviors. Overall, spatiotemporal patterns of most suitable foraging and resting habitat were similar, with shallow (10–15 m deep) nearshore (5–10 km from the coast) waters most suitable for both behaviors; however, habitats nearest to the coast (<5 km) were more suitable for foraging than resting. Overall, for foraging and resting, as water level increased turtles selected increasingly nearshore habitats where current speed was low and more variable direction. Overlap between most suitable habitats and current spatial zoning at the study site varied both seasonally and with water level, likely reflecting strong tidal influence on distribution and hence highlighting considerable opportunity for dynamic management. Our approach facilitates mechanistic insight into habitat selection and is generalizable across behaviors, taxa, and study systems, advancing the application of biologging tools to enhance the utility of HSMs and providing crucial context for decision‐makers in threatened species management.

## INTRODUCTION

Effective management of threatened species relies on identifying important habitats and managing threatening anthropogenic activities within them (Watson et al., 2014). Predictive statistical models, such as species distribution models (SDMs) and habitat suitability models (HSMs; hereafter collectively referred to as HSMs), have become essential decision‐making tools used by conservation planners in this manner (Franklin et al., 2011; Guisan et al., 2013; Welch et al., 2020). Such models link species occurrence (or presence) data, typically derived from direct sightings, camera trapping, and telemetry methods, to in situ environmental data (Elith & Leathwick, 2009; Hirzel & Le Lay, 2008). This has enabled identification of the location of suitable and unsuitable habitats, characterization of species–environment interactions and drivers of habitat selection, and the assessment of potential exposure or response to threats (Abrahms et al., 2019; Camprasse et al., 2017; Chambault et al., 2021; Hooker et al., 2011; Scales et al., 2016; Yorio et al., 2010).

Species may select distinct habitats according to resource requirements and competing demands associated with different behaviors; therefore, certain habitat features may be important for one behavior but not necessarily another (Frans et al., 2018; Picardi et al., 2022; Wilson et al., 2012). Black woodpeckers *Dryocopus martius* select disparate habitats for foraging and nesting, and selection for land‐cover types by Pumas *Puma concolor* was confounded by behavioral state (Brambilla & Saporetti, 2014; Zeller et al., 2014). If habitat selection is behavior‐specific, then by extension habitat suitability is also behavior‐specific, and failing to consider behavior in HSMs may result in inaccurate predictions of suitable habitat (i.e., extent under or overestimated), as well as offer limited mechanistic understanding of the drivers of habitat selection (Beumer et al., 2023; Beyer et al., 2010; Lundy et al., 2012; Wilson et al., 2012).

Exposure to and/or impact of threats could also be behavior‐specific; for instance, orcas *Orcinus orca* were more susceptible to disturbance by vessel traffic during feeding compared to resting, socializing, or traveling (Ashe et al., 2010), while predation risk for black‐tailed deer *Odocoileus hemionus columbianus* is higher when grazing in open pastures compared to resting in sheltered forests (Bose et al., 2018). Such examples demonstrate how ensuing management interventions to reduce the potential impact of threats may be rendered less effective if decision‐making is driven by HSMs that overlook behaviors. Finally, behaviors may contribute differently to individual fitness and growth (e.g., gaining energy by foraging, conserving energy by resting; Krebs & Davies, 2009). As such, management targeted toward behaviors that have the greatest impact on these population‐level processes, and by extension the habitats that support them, may significantly improve conservation outcomes (Roever et al., 2014).

Despite the clear importance of accounting for fine‐scale behaviors during conservation planning, obtaining spatially explicit behavioral data from free‐ranging animals remains challenging (Beyer et al., 2010; Wilmers et al., 2015; Wilson et al., 2012). Presence data typically lack detailed behavioral context; therefore, previous attempts to incorporate behavior into habitat suitability modeling have generally used relatively naïve definitions of behavioral states, such as encamped versus exploratory, migratory versus non‐migratory, or active versus inactive, typically inferred from advanced multi‐state movement‐based analyses of telemetry data (e.g., Step Selection Functions, Clustering, Hidden Markov Models, State Space Models; Klappstein et al., 2023; Picardi et al., 2022; Prima et al., 2022; Roever et al., 2014; Van Moorter et al., 2010). Such relatively coarse perspectives and putative allocations of behavior are especially common for aquatic taxa largely due to the challenges associated with data transmission through water rather than air. For instance, habitats suitable for foraging by Ross seals *Ommatophoca rossii* and pygmy blue whales *Balaenoptera musculus brevicauda* were identified and characterized across broad spatial scales by inferring subsurface foraging behavior from two‐dimensional surface location data (Area Restricted Search; Ferreira et al., 2024; Wege et al., 2021).

Recent guidelines from Northrup et al. (2022) highlight that integrating high‐resolution location data with information from other animal‐borne sensors can enhance our ability to make behavioral inferences and thus refine our understanding of habitat selection and suitability via deeper mechanistic insights (sensu Abrahms et al., 2016; Adam et al., 2019; Bose et al., 2018; Löttker et al., 2009; Scharf et al., 2016). For example, three distinct behaviors (traveling, running, and resting) by free‐ranging African dogs *Lycaon pictus* were differentiated by combining presence locations from a GPS with activity metrics from a tri‐axial accelerometer and gyroscope. In turn, this revealed habitat selection patterns that were not apparent from presence‐only data: distinct selection of roads for traveling but avoidance when resting (Abrahms et al., 2016). The importance of habitats nearby humans for feeding by African lions *Panthera leo* was only revealed when fine‐scale behaviors were explicated, again by associating high‐resolution GPS locations to accelerometer data (Suraci et al., 2019). Indeed, contemporary multi‐sensor biologging devices, particularly those incorporating video systems, confer the greatest possible insight into free‐ranging animal behaviors, especially for highly cryptic aquatic species (Heaslip et al., 2014; Jeantet et al., 2020; Nathan et al., 2022; Sato, 2020). Yet, despite representing a significant advance for the efficacy of HSMs and subsequent conservation outcomes, multi‐sensor biologging approaches remain underutilized in this context.

One aquatic taxon that stands to benefit from such investigations is sea turtles, with all seven extant species facing numerous threats across their complex life history (Wallace et al., 2011). Habitat suitability for sea turtles has been modeled over broad spatial scales to identify potential migratory routes and nesting, inter‐nesting, and foraging sites, yet these “behaviors” are more analogous to phenological stage (Dunkin et al., 2016; Fuentes et al., 2020; Fujisaki et al., 2020; Hawkes et al., 2007; Mancino et al., 2022; Marshall et al., 2020; Thums et al., 2017; Whittock et al., 2016b). At sea, within foraging sites, habitat suitability remains poorly understood, despite high residency and supporting population‐level processes and persistence (Shimada et al., 2016). The few studies that have addressed habitat suitability within a foraging site were based on sighting data from scuba‐ or boat‐based surveys, or acoustic and satellite telemetry, and did not consider fine‐scale behavior (DiMatteo et al., 2022; Selby et al., 2019; Wright et al., 2022). Since sea turtle foraging sites are commonly situated in coastal areas that tend to overlap with multiple anthropogenic pressures (Almpanidou et al., 2022; Hart et al., 2018), understanding behavior‐specific habitat suitability at foraging sites represents a crucial step toward improving conservation efforts for sea turtles.

Here, we employ a multi‐sensor biologging approach to incorporate fine‐scale behaviors into habitat suitability modeling, using the “data‐deficient” flatback turtle *Natator depressus*, a vulnerable species endemic to Australia (IUCN, 1996), as a case study. Using supervised machine learning, we predict habitats suitable for key in‐water foraging and resting behaviors at a coastal foraging site from high‐resolution motion sensor data, combined with GPS data and ancillary animal‐borne video footage matched to environmental features. We quantify the influence of environmental features on behavior‐specific habitat suitability and gain novel detailed insight into the mechanisms driving behavior‐specific habitat selection at a highly dynamic, macrotidal environment. Finally, we demonstrate the applied utility of our approach for decision‐makers by contextualizing the most suitable foraging and resting habitats within the current spatial zoning of the study site.

## MATERIALS AND METHODS

Animal use was approved by Animal Ethics Committees at Murdoch University (653‐R3164/19) and the Western Australian Government Department of Biodiversity, Conservation & Attractions (2016‐18/2019‐12‐B), licensed in accordance with relevant legislation by the Western Australian Government Department of Biodiversity, Conservation & Attractions (TFA 2019‐0042 and 08‐009604‐1) and the Western Australian Government Department of Primary Industries and Regional Development (U6‐2017‐2019/2020‐2022).

### Study site and data collection

**FIGURE 1:**
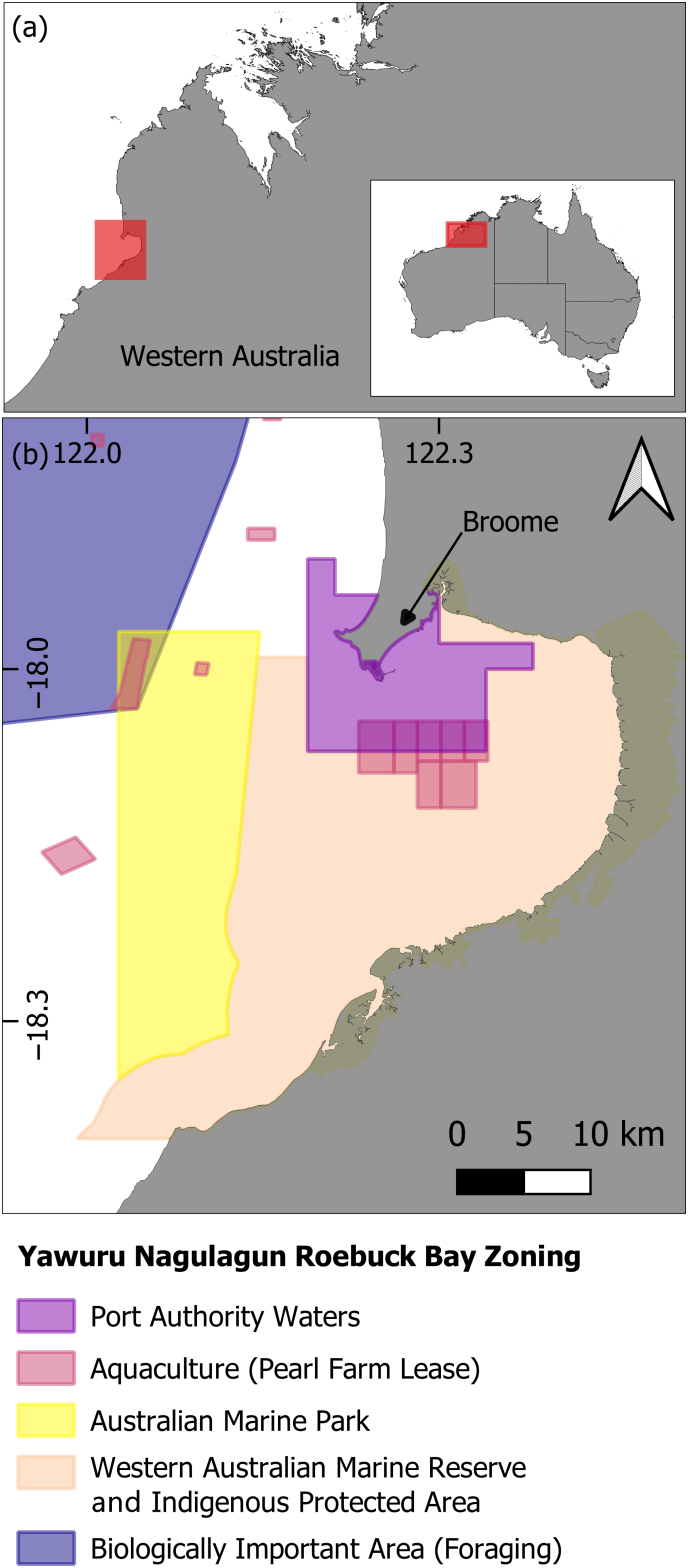
Location of (a) the study site, Yawuru Nagulagun Roebuck Bay in the western Kimberley region of Western Australia, and (b) the current spatial zoning.

The study site, Yawuru Nagulagun Roebuck Bay (hereafter YNRB) in the Kimberley region of Western Australia (Figure 1a), is a subtropical macrotidal coastal embayment (maximum spring tidal range ~10.6 m; Bennelongia 2009) and a year‐round foraging site for flatback turtles (Hounslow et al., 2022; Peel et al., 2024). Between 2018 and 2021, 51 flatback turtles were captured at sea and equipped with either a CATS‐Cam (multiple high‐resolution sensors including animal‐borne video camera) or a CATS‐Diary (multiple high‐resolution sensors, no camera; Customised Animal Tracking Solutions, CATS; Queensland, Australia). Full details for turtle capture and tag programing, deployment, and retrieval are available in Hounslow et al. (2022, 2023a).

### Behavior‐specific habitat suitability modeling

#### Behavior‐specific presence data

Geolocated behavior‐labeled dives, output from Hounslow et al. (2023a), were used as behavior‐specific presence data input into the HSMs in this present study. For full details describing the behavior‐specific presence dataset, please follow methods described in Hounslow et al. (2023a) using data provided in Hounslow et al. (2023b). Here, we briefly summarize these methods. For each individual CATS‐Diary and CATS‐Cam deployment, dives were first isolated from the continuous time‐depth record (collected at 1‐Hz sampling rate) and then assigned binary labels for the presence or absence of both benthic foraging and benthic resting based on observations from ancillary video when available. Then, for each behavior, dive‐phase‐specific features characterizing kinematic and two‐ and three‐dimensional aspects of behavior were calculated from metrics derived from the concurrently recorded high‐resolution tri‐axial motion sensor data (i.e., accelerometer and magnetometer). Motion sensor data were recorded at a sampling rate of 20–50 Hz, above the required minimum sampling rate (at least twice the dominant stroke rate, Nyquist–Shannon theorem; Shannon, 1949) and sufficient for capturing potentially complex movements with high signal variation. Derived metrics included mean overall dynamic body acceleration (ODBA; a common proxy for locomotory activity level; Gleiss et al., 2011), mean pitch angle (representing body posture), and variance in travel path heading (degree of tortuosity) for the descent, bottom, and ascent dive phases, as well as duration for all dive phases (including post‐dive surface interval) and depth variation during the bottom phase (for specific details, follow calculations in Hounslow et al., 2022, and see Hounslow et al., 2023a). The behavior‐labeled dive features were then used to train highly performant supervised machine learning algorithms to automatically detect the presence or absence of both foraging and resting from the same dive features calculated for all dives, including those dives without ancillary video (i.e., all CATS‐Diary deployments and CATS‐Cam deployments during periods when the camera was off). All behavior‐labeled dives were then geolocated (assigned geographical coordinates) via georeferenced dead‐reckoning (described in full in Hounslow et al., 2023a).

To model behavior‐specific habitat suitability in this study, the behavior‐labeled geolocated dives (output from Hounslow et al., 2023a) were treated as two separate presence datasets: one dataset for foraging presence and another dataset for resting presence. To account for temporal patterns of behavior‐specific habitat use by flatback turtles at the study site previously observed in Hounslow et al. (2023a), the foraging and resting presence datasets were both subset according to season (summer: October to March and winter: April to September) and hourly water‐level category as a result of tide height (Low <4 m, Mid 4–7 m, and High >7 m [from hourly water level height above tide gauge zero], Australian Government Bureau of Meteorology, 2022). This resulted in 12 separate presence datasets (*n*_Forage_ = 6, *n*_Rest_ = 6; Table 1).

**TABLE 1:** Geolocated behavior‐labeled dives (*n* = 4606) from Hounslow et al. (2023a) used as behavior‐specific presence data for behavior‐specific habitat suitability models for flatback turtles at Yawuru Nagulagun Roebuck Bay, Western Australia, subset according to season and water level categories attributed to tide height (Low < 4 m, Mid 4–7 m, and High > 7 m).

Season	Water level	Behavior‐specific presence data (*n*_dives_)	
		Forage	Rest
Summer	Low	335	128
	Mid	419	253
	High	319	227
Winter	Low	327	229
	Mid	1012	914
	High	246	197
Total		**2658**	**1948**

#### Environmental variables

Environmental variables describing the bathymetry, slope, aspect, and ruggedness of the sea floor, distance to the coast, and current speed and direction (*n* = 10; Table 2) were prepared as raster layers for the spatial extent of the study using the *Raster*, *ncdf4*, and *circular* R packages (Agostinelli & Lund, 2022; Hijmans, 2022; Pierce, 2023). To capture the broad range of current conditions at any given location within the macrotidal study site, current speed and direction were first calculated from current velocity data (flow components *u* and *v* along east and north), simulated over each hour of a full spring–neap tidal cycle using a custom depth‐averaged hydrodynamic model (Delft3D‐FLOW version 4.04.01; see Appendix S1: Section S1 for further details). Variables including mean, minimum, maximum, and variance of current speed and the circular variance of current direction were then calculated as separate raster layers for each water‐level category (Low, Mid, and High). All environmental variable raster layers were set to the same geographic datum (WGS84/EPSG: 4326), spatial extent (121.94040, 122.42500, −18.34166, −17.86563; Figure 1b), and grid cell resolution (30 m; the finest resolution of available environmental variable data, resampled by nearest neighbor interpolation if required). In total, 10 stacked raster layers were prepared for each water‐level category (Low, Mid, and High; Appendix S1: Figures S2–S4).

**TABLE 2:** Environmental variables prepared as raster layers for use in behavior‐specific habitat suitability models for flatback turtles at Yawuru Nagulagun Roebuck Bay, Western Australia.

Variable	Description	Source
DEM	Digital elevation from mean sea level indicative of bathymetry (m)	North West Shelf Bathymetry Digital Elevation Model (DEM) 2020 30 m (Lebrec et al. 2021); downloaded from Geoscience Australia Portal https://portal.ga.gov.au/, Commonwealth of Australia
SLOPE	Slope of sea floor in degrees (°)	Derived from DEM layer using *terrain* and *distance* functions from the *raster* package in R (Hijmans, 2022)
ASP	Orientation of slope in degrees from North (°)	
TRI	Terrain Ruggedness Index; topographic heterogeneity/roughness of seabed ranging from 0 to 1 (Riley et al., 1999)	
DIST	Minimum proximity distance from each grid cell centroid to the nearest coastline (km)	
Mean_SP	Mean current speed (m s^−1^)	Speed and direction statistics calculated from hourly water‐level data available from http://www.bom.gov.au/oceanography/projects/abslmp/data/index.shtml and simulated hourly current velocity data (Delft3D Flow hydrodynamic model^a^), then stacked for each water‐level category (Low < 4 m, Mid 4–7 m, and High > 7 m) Circular variance of current direction calculated using R package *circular* (Agostinelli & Lund, 2022)
Min_SP	Minimum current speed (m s^−1^)	
Max_SP	Maximum current speed (m s^−1^)	
SD_SP	Variance (SD) of current speed (m s^−1^)	
Var_DR	Circular variance of current direction, where 0 = unidirectional linear currents (low variance) and 1 = tortuous currents (high variance)	

^a^ See Appendix S1: Section S1 for Delft3D hydrodynamic model details.

#### Habitat suitability modeling

To predict the location and describe the characteristics of the most suitable habitats for in‐water foraging and resting by flatback turtles, we implemented a series of random forest (RF) HSMs (Breiman, 2001). RF is a popular supervised machine learning algorithm for predicting ecological processes, consistently outperforming other algorithms for modeling habitat suitability (Chambault et al., 2021; Li et al., 2017; Stupariu et al., 2022). Comprising an ensemble of decision trees, each tree in an RF predicts the response variable from a random subset of predictor variables. RFs are insensitive to collinear data and avoid overfitting, and the random decision process allows for robust predictions from repeated measures such as our behavior‐specific presence data (Hastie et al., 2009; Liaw & Wiener, 2002).

For each behavior, separate RF HSMs (*n*_Forage_ = 6, *n*_Rest_ = 6) were developed in the *caret* R package (Kuhn, 2021), using the behavior‐specific presence dataset as the response variable. To create a binary response for each behavior‐specific presence dataset and also to characterize the environmental conditions across the full extent of the study site, background data were randomly generated in equal proportion to the behavior‐specific presence data in each dataset using the *Raster* package (Hijmans, 2022; Hijmans et al., 2021; Phillips et al., 2009). An equal proportion of presence‐background data is considered appropriate for small datasets (Barbet‐Massin et al., 2012), and an equal proportion of presence‐background data also reduces the potential for predictive bias caused by imbalanced datasets (Chen et al., 2004). For each RF HSM, the environmental variable data (*n* = 10) were extracted at the presence‐background locations (Appendix S1: Figure S5), using the *raster* package in R (Hijmans, 2022) and used as predictor variables.

For each RF HSM, input data (response and predictor variables) were split randomly for model training (80%) and testing (20%). All RF HSMs were trained with *ntree* (number of trees) set at 1000 and tuned by hyperparameter *mtry* (number of predictor variables randomly selected at each tree) via internal 10‐fold cross validation (*k* = 10, with 10 repeats). Model performance was evaluated by fitting each trained RF HSM to the corresponding withheld test dataset and then calculating the area under the receiver operating curve (AUC) using the R package *pROC* (Robin et al., 2011). AUC scores range from 0 to 1, where 1 represents a model with perfect predictive ability and 0.5 represents poor predictive ability equal to random chance (Fourcade et al., 2018). Habitat suitability for each behavior was predicted across the entire extent of the study area as a habitat suitability index (HSI; 0–1, with higher values indicating areas of more suitable habitat). Each trained RF HSM was fitted separately to the stacked environmental variables corresponding to the correct water‐level category, using the *predict* function in the *raster* package. The most suitable habitats were determined using an HSI threshold >0.5 (Austin et al., 2019). In addition to predicting HSI for each behavior, season, and water‐level category, seasonal mean HSI across all water‐level categories was also calculated, with HSI for each water‐level category (Low, Mid, and High) weighted to represent the proportion of hourly water‐level data within each water‐level category (0.24, 0.52, and 0.24, respectively). Finally, key environmental features were identified via importance scores for each environmental variable (scaled to between 0 and 100) calculated using the *caret* R package, and their influence on habitat suitability for both behaviors was assessed via marginal effects plots created using the *pdp* R package (Greenwell, 2022; Kuhn, 2021).

### Overlap with spatially managed zones

Application of this approach was demonstrated by calculating the proportion of the most suitable foraging and resting habitat (HSI > 0.5) overlapping spatially managed zones at the study site (Figure 1b). Overlap for each behavior, season, and water‐level category was calculated for Australian Marine Parks and Western Australian Marine Reserves: Roebuck Marine Park and YNRB Marine Park. Because the Yawuru Indigenous Protected Area Sea Country falls within the spatial boundary of the Western Australian Marine Reserve, both zones were treated as one for the purpose of calculating overlap with suitable foraging and resting habitats. Overlap was also calculated for the Biologically Important Area for foraging by flatback turtles, as well as areas zoned for commercial or industrial use: Kimberley Port Authority waters supporting the Port of Broome and aquaculture leases *Pinctada maxima* pearl farms (for access information for all external datasets, refer to *Data availability statement*). All analyses were performed using R version 4.3.0 (R Core Team, 2020), and all maps were created using the *tmap* package (Tennekes, 2018), except visualizations in Figure 1 which were prepared using QGIS version 3.28.10 (https://qgis.org/).

## RESULTS

In total, 4606 geolocated behavior‐labeled dives by 42 adult flatback turtles tagged between 2018 and 2021 (Appendix S1: Table S1; data from Hounslow et al., 2023a) were used as behavior‐specific presence data for habitat suitability modeling (Table 1, Appendix S1: Figure S5).

### Behavior‐specific habitat suitability modeling

**FIGURE 2:**
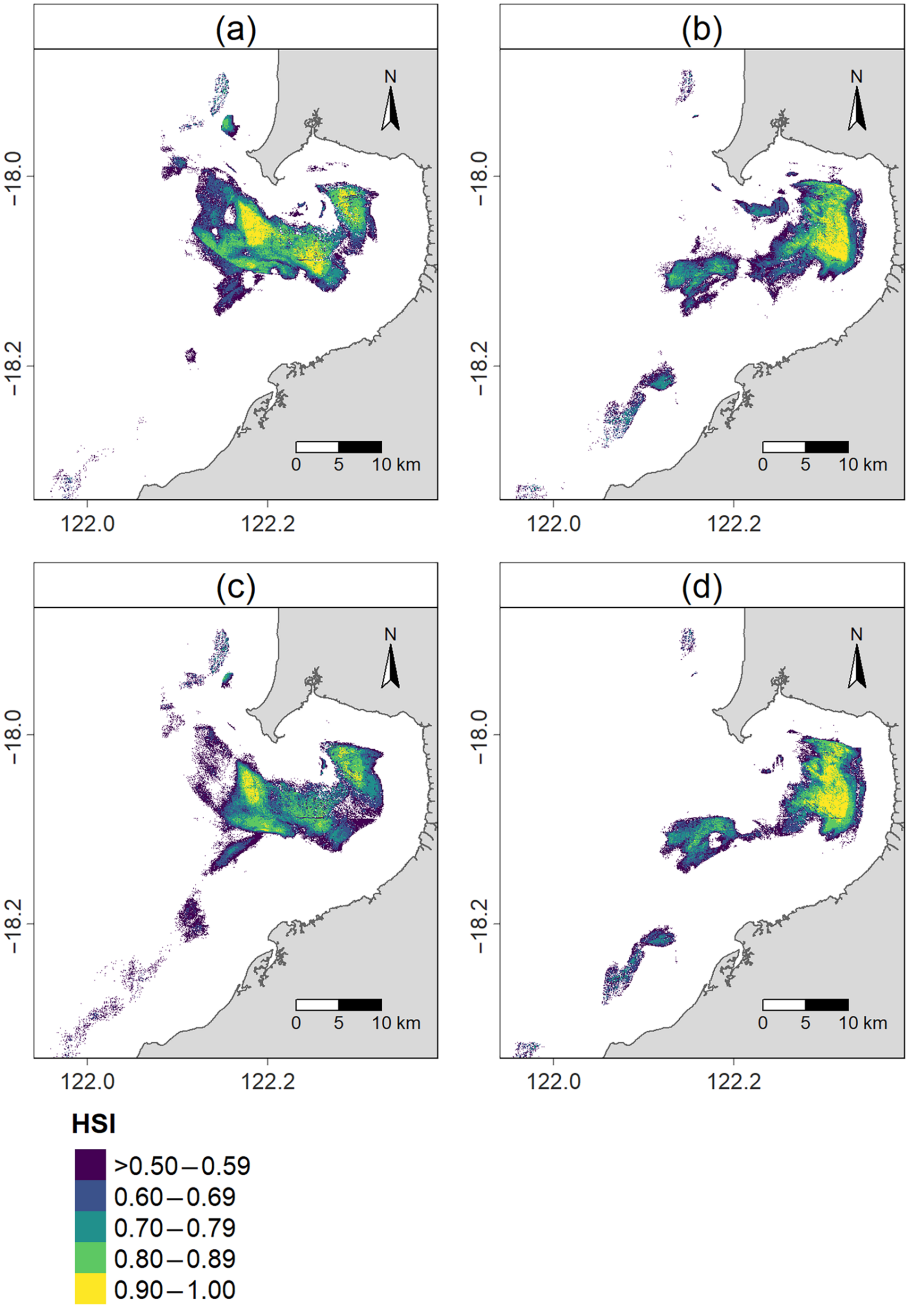
Behavior‐specific habitat suitability for flatback turtles at Yawuru Nagulagun Roebuck Bay, Western Australia. Predicted areas of most suitable in‐water foraging habitat during (a) summer and (b) winter and suitable in‐water resting habitat during (c) summer and (d) winter. Habitat suitability index (HSI) shows most suitable habitats (HSI > 0.5) and is presented as the seasonal mean of random forest model predictions across all water level categories (attributed to tide height; Low < 4 m, Mid 4–7 m, and High >7 m), weighted according to the proportion of hourly water level data in each water level category. Unsuitable habitats are colored white.

The RF HSMs (*n*_Forage_ = 6, *n*_Rest_ = 6) performed with AUC scores ranging from 0.84 to 0.98 (Appendix S1: Figure S6), representing good (AUC > 0.8) or excellent (AUC > 0.9) predictive ability (Fourcade et al., 2018). Qualitatively, there appeared to be minimal spatial variation between habitats predicted as most suitable (HSI > 0.5) for flatback turtle foraging (Figure 2a,b) and resting (Figure 2c,d). However, the location of the most suitable habitat for both behaviors varied temporally, characterized by an inshore–offshore shift according to rising and falling water levels that were attributed to the tide. This pattern was more pronounced during winter than summer (Figure 2, Appendix S1: Figures S7 and S8). Areas nearest to the eastern shore, the deepwater channel directly adjacent to the northern headland, and offshore areas were generally unsuitable for both foraging and resting (Figure 2).

### Key environmental features and influence on behavior‐specific habitat suitability

**FIGURE 3:**
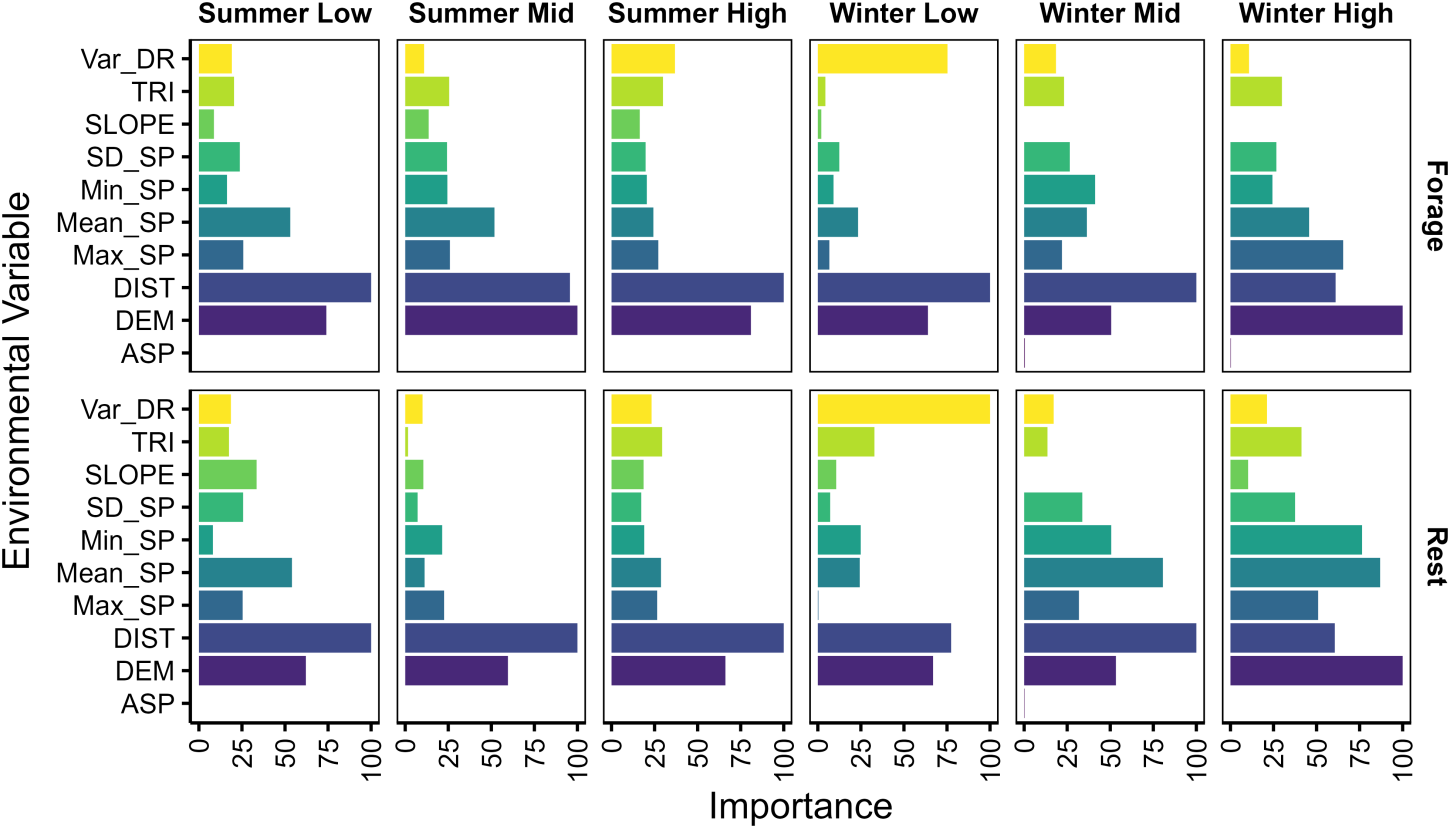
Variable importance for random forest models determining habitat suitability for in‐water foraging and resting by flatback turtles, derived from multi‐sensor biologging data. Panel columns indicate season and water level category, and panel rows indicate behavior. Variable importance scores are scaled to 0–100; higher values indicate contribution to reduced error rate and more important variables. Variable names are described in Table 2.

The similarity observed in the distribution of suitable foraging and resting habitat (Figure 1) was further supported by the same environmental variables best determining habitat suitability for both behaviors (Figure 3). Distance from the coast (DIST) and bathymetry (DEM) were the predominant environmental variables influencing habitat suitability for both foraging and resting, with high importance scores (>50) across all 12 RF HSMs (water‐level categories, seasons, and behaviors). Features related to currents were moderately important predictors of suitable foraging and resting habitat, in particular mean current speed (Mean_SP) and the variance in current direction (VAR_DR) (Figure 3).

**FIGURE 4:**
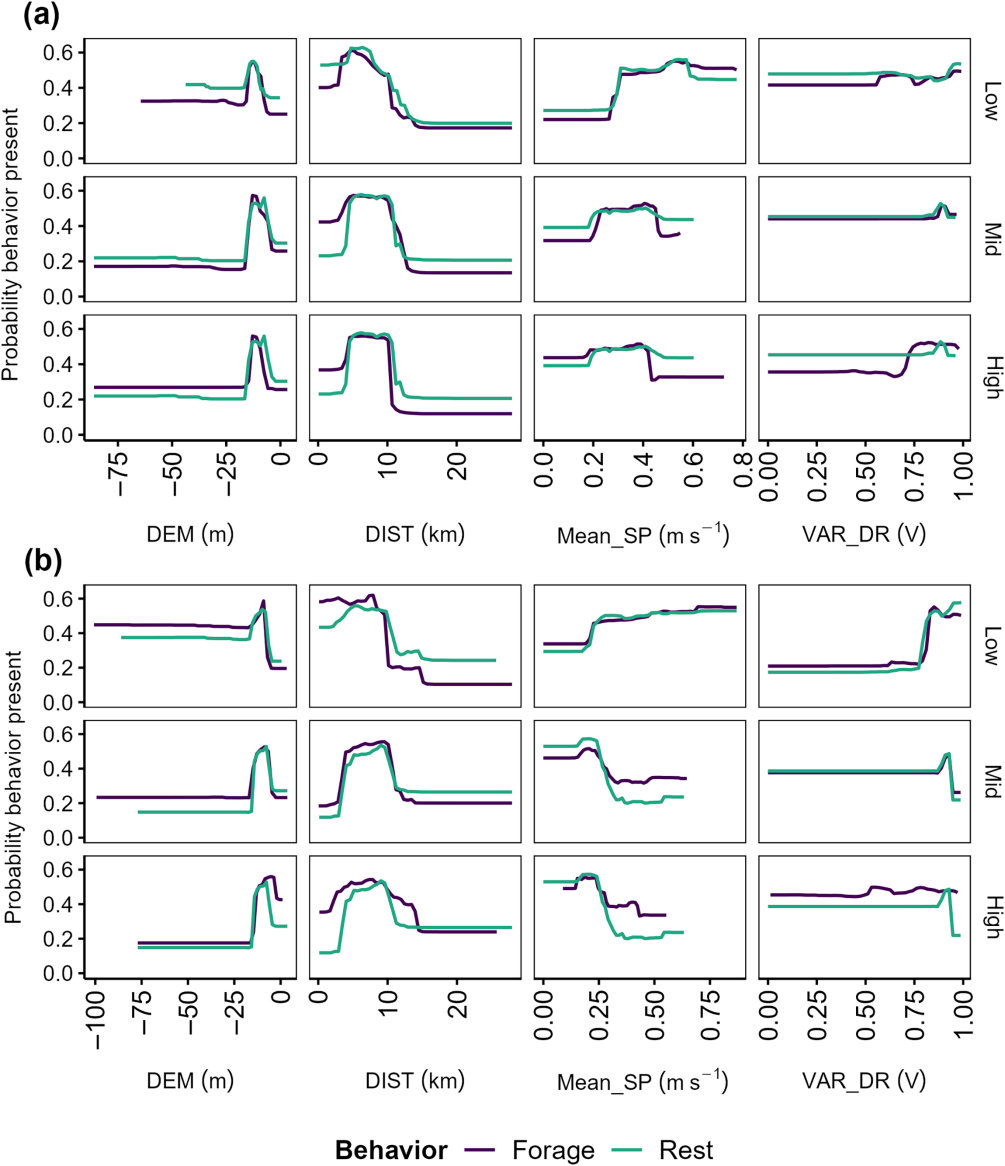
Response curves showing marginal effects of key environmental variables on the presence probability for in‐water foraging and resting by flatback turtles during (a) summer and (b) winter at Yawuru Nagulagun Roebuck Bay, Western Australia, as predicted by random forest habitat suitability models (0 = low probability, 1 = high probability). Panel rows indicate water level categories attributed to tide height: Low (<4 m; top row), Mid (4–7 m; middle row), and High (>7 m; bottom row). See Table 2 for environmental variable descriptions.

Combined with maps of the most suitable habitats (HSI > 0.5; Figure 2), marginal effects for these key features showed that the most suitable foraging and resting habitats for flatback turtles were nearshore (~5–10 km from the coast) and characterized by shallow bathymetry (~10 m deep; Figure 4). There was a steep decline in the probability of habitat being suitable for both foraging and resting at locations >10 m deep and >10 km from the coast. Notably, despite the spatiotemporal distribution of suitable habitat appearing similar for foraging and resting, some distinctions were observed. For instance, habitats nearest to the coast (<5 km) were generally more suitable for foraging than for resting (Figure 4). In addition, at those nearshore locations (<5 km), deeper bathymetry (>15 m) habitats were better suited for foraging than resting—particularly during winter; however, the probability of waters >50 m deep being suitable for either behavior was between 20% and 40% depending on water level (Figure 4).

Other environmental features influencing the presence of both behaviors were current speed (Mean_SP) and variance in current direction (VAR_DR; Figure 4). Foraging and resting were more likely to occur at locations with low current speed (<0.4 m s^−1^) when the water level was mid (4–7 m) or high (>7 m; Figure 4b). However, when the water level was low (<4 m), locations where current speeds were faster were more suitable for both foraging and resting (Figure 4). Overall, turtles were also more likely to forage and rest at habitats where current direction was more variable (VAR_DR >0.5; Figure 4b).

### Overlap between suitable habitats and spatial zoning

**FIGURE 5:**
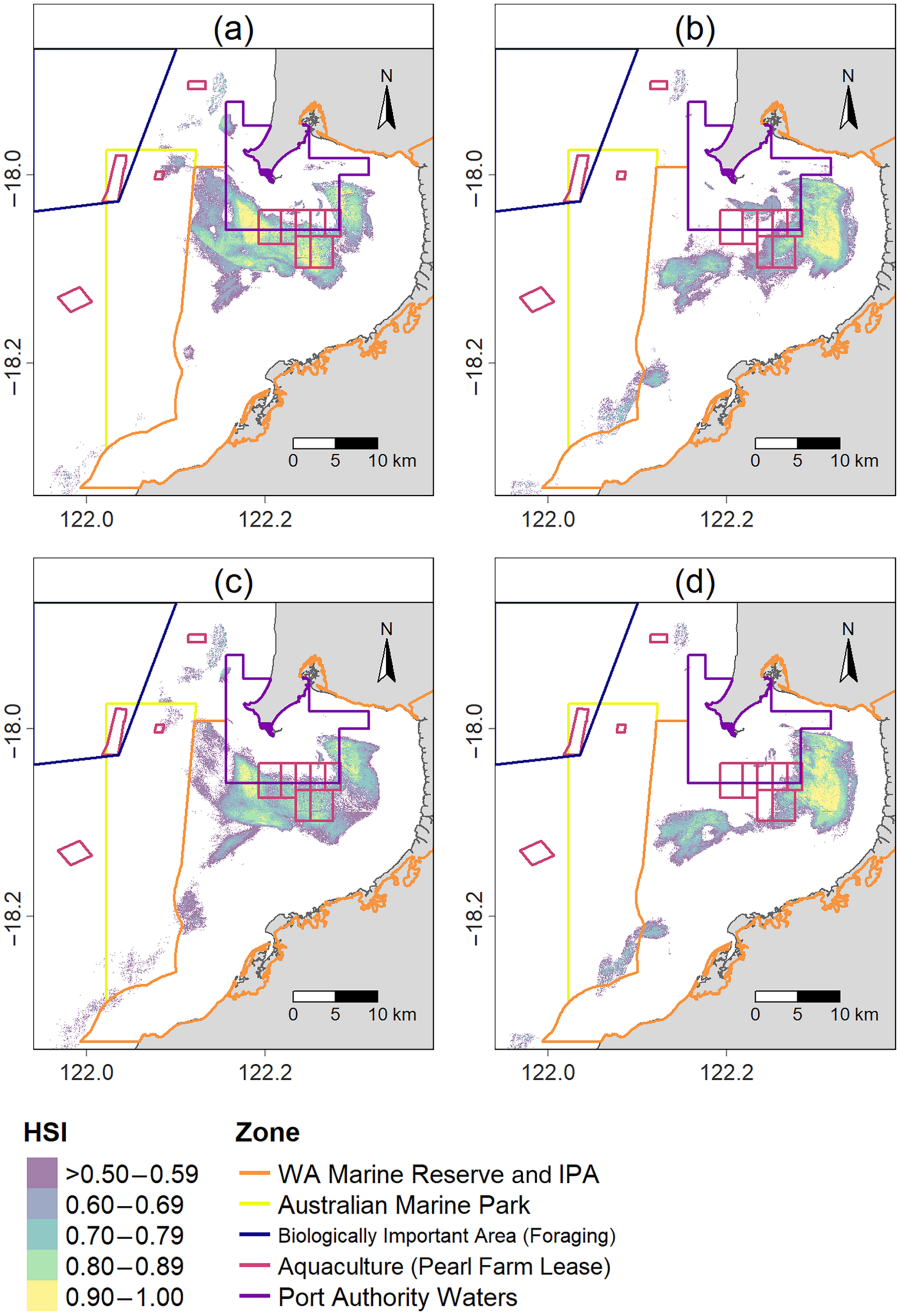
Behavior‐specific habitat suitability for in‐water foraging during (a) summer and (b) winter and in‐water resting habitat during (c) summer and (d) winter by flatback turtles at Yawuru Nagulagun Roebuck Bay, Western Australia, overlaid with spatially managed zones (also see Figure 1b). Habitat suitability index (HSI) shows most suitable habitats (HSI > 0.5) and is presented as the seasonal mean of random forest model predictions across all water level categories (attributed to tide height; Low <4 m, Mid 4–7 m, and High >7 m), weighted according to the proportion of hourly water level data in each water level category. Unsuitable habitats are colored white.

The proportion of the most suitable foraging and resting habitat (HSI > 0.5) for flatback turtles occurring within designated marine reserves varied. While spatial overlap with Roebuck Marine Park offshore was generally low (<11%), a consistently high proportion (~46%–90%) of most suitable habitat for both behaviors occurred within the boundaries of YNRB Marine Park and Yawuru IPA located further inshore. This overlap increased as water level (attributed to tide height) increased and was highest during winter (Figure 5 and Table 3). Conversely, the proportion of the most suitable foraging and resting habitat overlapping commercial and industrial use zones was highest during summer and increased as water level decreased (Figure 5 and Table 3). For instance, when water level was low (<4 m), up to ~26% of the most suitable foraging habitat was located within Port Waters servicing the Port of Broome. The proportion of the most suitable foraging and resting habitat inside aquaculture leases, which are located within both port and YNRB marine park boundaries, was generally lower (maximum 20%), particularly during winter when water level was high (>7 m; <3%; Table 3).

**TABLE 3:** Proportion of habitat predicted as most suitable (HSI > 0.5) for in‐water foraging and resting by flatback turtles within spatially managed zones at Yawuru Nagulagun Roebuck Bay, Western Australia, according to season and water‐level categories attributed to tide height (Low <4 m, Mid 4–7 m, and High >7 m).

Behavior	Season	Water level	WA Marine Reserve and IPA (%)	Australian Marine Park (%)	BIA (%)	Port Authority Waters (%)	Aquaculture (pearl lease) (%)
Forage	Summer	Low	53.09	10.19	0.0	26.15	20.43
		Mid	74.97	2.93	0.0	18.25	16.89
		High	76.47	2.45	0.0	15.05	20.18
	Winter	Low	48.20	10.72	0.0	17.85	8.91
		Mid	78.10	5.56	0.0	13.90	16.96
		High	89.93	1.47	0.0	7.62	3.07
Rest	Summer	Low	47.82	13.76	0.0	22.17	16.34
		Mid	77.20	7.05	0.0	10.72	14.14
		High	77.57	3.90	0.0	11.96	17.50
	Winter	Low	54.77	14.11	0.0	13.19	10.72
		Mid	84.59	7.85	0.0	4.92	10.36
		High	88.08	7.19	0.0	7.34	2.18

*Note*: Since zones may overlap (Figure 1b), suitable habitat can occur in multiple zones; therefore, percentages do not add up to 100.

Abbreviations: BIA, Biologically Important Area (Foraging); IPA, Indigenous Protected Area.

## DISCUSSION

This study is one of the first to use a multi‐sensor biologging approach to incorporate fine‐scale behavior in the context of modeling habitat suitability (but for terrestrial examples, see Abrahms et al., 2016; Suraci et al., 2019). We developed high‐performing behavior‐specific HSMs for flatback turtles (AUC ≥0.84), using high‐resolution (50 Hz) motion sensor data from tri‐axial accelerometers and magnetometers, validated by ancillary animal‐borne video footage and combined with GPS data. In doing so, we provide the first quantitative analysis of behavior‐specific habitat suitability for any marine organism using multi‐sensor biologging data, to our knowledge. Moreover, we describe the spatiotemporal distribution of habitat most suitable for in‐water foraging and resting by a species of sea turtle within a resident foraging site, which remains a relatively understudied aspect of sea turtle ecology (Hamann et al., 2010; Hays, 2008). By characterizing these habitats, we gain insight into the drivers of habitat selection for the vulnerable and data‐deficient flatback turtle (Commonwealth of Australia, 1999; IUCN, 1996), which will prove important for local conservation managers and decision‐makers. More broadly, our method to identify and characterize suitable habitat is transferable to a range of distinct behaviors, other taxa, and other study systems (e.g., terrestrial and aquatic), representing a significant advance in the efficacy of multi‐sensor biologging combined with HSMs as a tool for conservation planning and threatened species management.

### Environmental drivers and behavioral suitability

Importantly, in this study, we were able to quantitatively demonstrate differences between suitable resting and foraging habitat, despite previously finding no major qualitative behavioral difference in the spatiotemporal distribution of flatback turtles foraging and resting within YNRB (Hounslow et al., 2023a). Our results support the premise that animals may often exhibit different behaviors in different areas because the same environmental features are not required for all behaviors, ultimately giving rise to fine‐scale differences in habitat suitability. For instance, habitat suitable for resting may be characterized by safe shelter, whereas suitable foraging habitat likely requires abundant food resources (Brambilla & Saporetti, 2014; Roever et al., 2014; Wilson et al., 2012). This demonstrates the power of combining multi‐sensor biologging with quantitative spatial analyses using machine learning for modeling and understanding habitat suitability from a mechanistic perspective.

Research into the drivers of inshore foraging behaviors was recently recognized as a management opportunity for flatback turtles (Peel et al., 2024). Here, bathymetry and distance from the coast emerged as the key environmental features of foraging and resting habitat suitability for flatback turtles, which are both well documented as significant predictors of sea turtle presence (Shaver et al., 2013; Whittock et al., 2016b). Generally, suitable foraging and resting habitat occurred in shallow (bathymetry < 15 m), nearshore (<10 km from the coast) habitats within the study site. This relatively narrow suitability could be related to sea turtle diving physiology, where turtles select dive depths that maximize diving efficiency (Hays et al., 2004). Additionally, shallow, nearshore locations are increasingly turbid at this macrotidal study site, potentially hindering detection by and offering protection from coastal predators such as tiger sharks *Galeocerdo cuvier*, which are known to prey on sea turtles including flatback turtles at this study site (Heithaus et al., 2008; Hounslow et al., 2021). It is also noteworthy that the main “channel” at YNRB is exceptionally deep (>80 m). Deeper waters were generally unsuitable for both behaviors, with minimal to no evidence of channel use according to the behavioral presence data (Appendix S1: Figure S5 read in conjunction with DEM panel in Appendix S1: Figures S2–S4). While the rest of the bay does have some smaller drainage channels that extend from intertidal creeks on the eastern shores, these channels are highly dynamic, relatively small in scale, and subsequently not picked up in the bathymetric data available across the entire study area.

Among the most striking differences in behavior‐specific habitat suitability was the increased probability of foraging occurrence in shallow habitats very near to the coast (<5 km), supporting evidence that flatback turtles primarily use intertidal areas for foraging and proximate subtidal areas for resting (Hounslow et al., 2023a). This may be due to a higher abundance of benthic prey items in intertidal areas, which in YNRB can be contained in highly productive mudflats (Pepping et al., 1999). Alternatively, it could also be the result of resting being either risky in intertidal areas where semi‐diurnal tidal movements can exceed 10 m on the largest spring tides and turtles may end up stranded on land or because dives in shallow water may simply be too short to allow turtles to effectively rest. As a result of lung regulated buoyancy control, shallow dives inadvertently result in short dive durations (Hays et al., 2004), which may defeat the purpose of resting in the first place.

In addition to bathymetry and distance from the coast, currents strongly characterized behavior‐specific habitat suitability for flatback turtles, which is not surprising given currents influence the spatial distribution of sea turtles at oceanic scales (Grüss et al., 2018) and also in light of the extent of the tidal range and currents that characterize YNRB. It has previously been established that sea turtles use currents in various ways within coastal foraging sites, including for transport and to increase foraging efficiency (Brooks et al., 2009; Senko et al., 2010). In general, habitat most suited to both foraging and resting occurred at locations where current direction was highly variable (VAR_DR > 0.75). By foraging and resting in these areas, turtles are presumably less at risk of being displaced compared to locations where currents are more directional. Turtles may still be present in areas of directional currents; however, individuals are likely performing other behaviors such as active swimming or traveling, as opposed to relatively stationary foraging or resting on the seafloor. Indeed, if highly directional currents were to have been selected at low water levels (<4 m) when the western, outer edge of YNRB is most suitable for both behaviors, turtles may be at risk of drifting into unsuitable deeper habitat further offshore. This lends further support for turtles selecting habitat where currents are more variable to avoid displacement while foraging and resting; locations where current direction is more variable may simply result in less passive advection, thus increasing residency of turtles in these areas. Interestingly, while low current speed (<0.25 m s^−1^) was preferred in general, when water level was low (<4 m), turtles selected areas where currents were faster (>0.25 m s^−1^) for both foraging and resting. Combined with more variable current direction, this suggests that turtles are not completely averse to increased current speed, providing it does not result in displacement to unsuitable foraging and resting habitat.

The spatial extent of this study was limited to a location where tidal currents are dominant, relatively strong, and thus simple to simulate; therefore, further research will be required to confirm the causative factors regarding current‐mediated behavior and habitat use by flatback turtles. Habitat suitability modeling for predicting the location of both inshore and offshore foraging areas has certainly taken broad‐scale ocean current patterns into account previously (Thums et al., 2017); however, the operational scale is presently not comparable to that of this study. It could be interesting to compare the response of flatback turtles to currents in YNRB to that at other foraging sites, including those at locations further offshore where the direct effects of tidal currents may be relatively small in comparison (Peel et al., 2024; Thums et al., 2017; Whittock et al., 2016a). We recommend the deployment of multi‐sensor biologging devices at sites with distinctly different current characteristics as a fruitful area of future research to better understand the behavioral plasticity of flatback turtles in response to currents. Even so, the experimental design of biologging studies can be associated with complex logistical, technological, theoretical, analytical, and financial challenges (Williams et al., 2020), representing a potential impedance to wider uptake in future research and a possible explanation for underutilization of biologging in this context to date.

While our HSMs performed exceptionally well, another caveat was that they did not consider all environmental variables that may be informative for predicting suitable habitats for sea turtles. Thus, our mechanistic insight into habitat selection may be described as relatively modest, despite its' novelty. For instance, at the larger continental scale, benthic geomorphology is an important predictor of flatback turtle foraging areas (Thums et al., 2017). Locally, within YNRB, various types of soft‐sediment habitats contain a plethora of soft‐bodied invertebrates, sea pens, and sea cucumbers that are typical of the diet of flatback turtles (Limpus, 2007; Pepping et al., 1999; Whittock et al., 2016a). Benthic substrate and community habitat data have been shown to impact habitat use by sea turtles (Fujisaki et al., 2016) and may serve as useful proxies of food resource availability and therefore astute predictors of suitable benthic foraging habitat. Although benthic monitoring has previously occurred in YNRB, these data are not currently available over the full extent of the study site or at spatial scales relevant to fine‐scale behaviors. Therefore, we recommend implementation of fine‐scale benthic monitoring programs in YNRB that will facilitate revisiting this analysis once such data become available. Other relevant environmental variables that were not included in this study are sea surface temperature, turbidity, pH, and chlorophyll (cf. DiMatteo et al., 2022). Such data would require a bespoke ecohydrological model since they are not available through remote sensing platforms at the highly resolved spatiotemporal scale of analysis relevant to fine‐scale behaviors. This limitation should directly motivate future research to collect additional ecohydrological data to ensure a more sagacious understanding of significant habitats for flatback turtles.

### Overlap with spatial zoning

In addition to delineating and characterizing foraging and resting habitat, we also demonstrated application of this approach by assessing the spatiotemporal overlap between suitable habitats and the current spatial zoning at YNRB. Depending on specific management priorities (e.g., reducing bycatch vs. mitigating vessel strikes), this information (when accessible) may be useful for future impact assessments and provides actionable support for management planning (e.g., marine park monitoring and zoning). Overall, the majority of habitat suitable for both foraging and resting by flatback turtles captured in YNRB was located within Western Australian Marine Reserves (YNRB Marine Park and Yawuru Indigenous Protected Area) between ~48% and 89% depending on water level attributed to the tide height. The effectiveness of marine reserve waters in protecting most suitable foraging and resting habitats for flatback turtles was most pronounced at higher water levels (>7 m), as turtles increasingly foraged and rested nearer to the coast. Despite not being devoid of threats, their relatively low prevalence in marine reserve waters results in large areas of suitable habitat not heavily impacted by human activity. As such, reserve design appears generally well‐placed to offer effective protection of suitable foraging and resting habitats for flatback turtles. However, when the water level was low (<4 m), up to approximately 26% of most suitable foraging and resting habitat was located within Port Authority Waters servicing the Port of Broome. Here, turtles are potentially exposed to multiple in‐water threats which may impact both foraging and resting behaviors, many directly or indirectly linked to vessel use: disturbance, injury and mortality from vessel strike, marine pollution (e.g., oil spills, entanglement with discarded fishing gear, and noise disturbance), and dredging (e.g., entrainment, habitat loss, and alteration) (Dickerson et al., 2007; Hazel et al., 2007; Roman et al., 2021; Wallace et al., 2011; Whittock et al., 2016b, 2017). For instance, dredging operations may irrevocably alter benthic habitats and reduce diversity and abundance of flora and fauna, both by direct physical damage and indirectly by changing water quality and/or turbidity (Wallace et al., 2011; Whittock et al., 2016b, 2017). Due to the predominantly infaunal diet of flatback turtles at YNRB (Hounslow et al., 2023a), flatback turtle foraging may be adversely impacted in Port Waters. However, turtles also use these waters for resting on the seafloor. Similarly, during the inter‐nesting period, flatback turtles preferentially used dredged areas for resting, and it is certainly possible that turtles' behaviors may be differentially affected by dredging (Whittock et al., 2017). Given suitable foraging and resting habitat for flatback turtles in YNRB is spatially restricted to these areas at low water levels (<4 m), the construction of a new deep water floating wharf at the Port of Broome (Kimberley Marine Support Base; www.kmsb.com.au) as well as a proposed recreational boat launching facility (Broome Boating Facility; www.broomeboatingfacility.com.au) within Port Waters is notable. In addition to potential short‐term impacts during construction, both developments are designed to increase operational capacity regardless of tide height (i.e., vessel loading, unloading, and launching) and as such will increase vessel traffic in the area and possibly increase associated threats faced by turtles using this area for resting and foraging. Further monitoring may be needed to quantify the impacts associated with this overlap.

Considerable suitable habitat—up to 20% for foraging in summer—also occurred inside waters zoned for commercial pearl leases. Here, turtles may be more exposed to anthropogenic threats including vessel strike, gear entanglement, and disturbance from underwater noise, although globally, incidents related to pearling operations are rare (Bath et al., 2023). Despite increased exposure to such threats, there have been no formal reports of flatback turtles becoming entangled in pearl lines or struck by pearling vessels within YNRB. Furthermore, pearl leases may offer predictable foraging opportunities for the flatback turtle, which follows a carnivorous diet including molluscs (Limpus, 2007; Wildermann et al., 2017; Zangerl et al., 1988). Anecdotally, local pearl operators suggest that this may be the case (Jenna L. Hounslow, personal communication), yet no records exist for sea turtles foraging directly on farmed pearl oysters, which are suspended mid‐water inside mesh panels. These panels were only visible once for one individual from over 109 h of animal‐borne video footage, and the turtle did not attempt to feed on them (Hounslow et al., 2023a). Indeed, if turtles were feeding on pearl oysters in suspended mesh panels, they would be utilizing a pelagic foraging mode. However, the foraging HSMs in this study only represent suitability for benthic foraging, suggesting that the mesh panels themselves play no direct role in the suitability of pearl leases. Instead, it is likely that the benthic habitat over which the pearl lines are suspended provides suitable foraging habitat, irrespective of commercial operations. Furthermore, similar to Port Waters, flatback turtles at YNRB are likely restricted to the vicinity of pearl leases for both foraging and resting because waters further offshore are generally unsuitable, intertidal habitats become inaccessible with decreasing water levels attributed to falling tide height, and waters of this depth are preferred for diving efficiently (~10–15 m deep) (Hays et al., 2004; Hounslow et al., 2022).

When combined with evidence of spatial overlap between foraging and resting areas in (Hounslow et al., 2023a, 2023b), we consider overlap with spatially zoned areas strongly influenced by temporal drivers, regardless of behavior. Dynamic management solutions, based on the temporal patterns in suitability observed in this study according to seasonal and/or diel forecasted tide height, may be effective management solutions in this context (Maxwell et al., 2015). Considering this further, overlap with spatial zoning would probably be similar if quantified from presence‐only spatial data (without behavioral data following a multi‐sensor approach); however, we reiterate that spatial data require a much higher degree of inference about habitat selection, resulting in potentially inadequate management recommendations. As such, it is compelling to note that no suitable foraging habitat for turtles captured in YNRB occurs within waters federally designated as a Biologically Important Area (BIA) for foraging by flatback turtles. This lends additional support to the management opportunities proposed by Peel et al. (2024), which include a call to extend the current BIA into YNRB.

## CONCLUSION

Our method to inform habitat suitability modeling with high‐resolution multi‐sensor biologging data is broadly transferable to other behaviors, taxa, and study systems. Despite some inherent challenges, biologging represents a valuable enhancement to the suite of practical tools used by conservation planners for threatened species management. We reiterate that enabling such targeted approaches to decision‐making, alongside such broad applicability, can make dynamic management even more powerful.

## AUTHOR CONTRIBUTIONS

Jenna L. Hounslow, Sabrina Fossette, and Adrian C. Gleiss conceived the ideas. Jenna L. Hounslow, Sabrina Fossette, Anton D. Tucker, and Scott D. Whiting collected data. Jenna L. Hounslow conducted analyses with input from Arnold van Rooijen. Jenna L. Hounslow led the writing of the manuscript. All authors contributed critically to the drafts and gave final approval for publication.

## CONFLICT OF INTEREST STATEMENT

The authors declare no conflicts of interest.

## Supporting information


Appendix S1.

## Data Availability

Data supporting this research are available following methods described in Hounslow et al. (2023a) at https://doi.org/10.1111/1365-2664.14438, using data available from Hounslow et al. (2023b) at https://doi.org/10.5061/dryad.7wm37pvzb. The resulting output (pre‐processed data; behavior‐labeled geolocated dives representing locations of foraging and resting by flatback turtles) is sensitive and not available publicly. These pre‐processed data (behavior‐labeled geolocated dives) are owned by Murdoch University and the Western Australian Government Department of Biodiversity, Conservation, and Attractions (DBCA), and available to qualified researchers by contacting DBCA's Northwest Shelf Flatback Turtle Conservation Program (NWSFTCP) Principal Research Scientist via email at turtles@dbca.wa.gov.au and requesting the dataset (a CSV file titled: “YNRB Flatbacks Geolocated behaviour labelled dives”) from the NWSFTCP YNRB Foraging Flatbacks Project. The pre‐processed dataset (behavior‐labeled geolocated dives) and all other external data (publicly available with access information described below) were processed according to Hounslow et al. (2025) with R code available in Figshare at https://doi.org/10.6084/m9.figshare.28039586. Hourly sea level data for Broome, Western Australia (Station Number BoM = 003102, ANTT = 62650, GLOSS = 040, UHSLC = 166), are from the Australian Baseline Sea Level Monitoring Project (ABSLMP), © National Tidal Centre; available at http://www.bom.gov.au/oceanography/projects/abslmp/data/index.shtml. Bathymetry data extracted for the study area extent and coordinate reference system (Lebrec et al., 2021; GeoTIFF raster, EPSG:4326‐WGS 84/nearest resampling method) can be downloaded from the Geoscience Australia Portal. Roebuck Marine Park boundary was extracted from the national dataset of Australian Marine Parks (© Commonwealth of Australia, Australian Government Department of Climate Change, Energy, the Environment and Water, 2023) using shapefile data available at http://www.environment.gov.au/fed/ (Title Australian Marine Parks). Yawuru Nagulagun Roebuck Bay Marine Park boundary (© Department of Biodiversity, Conservation and Attractions, 2023) shapefile data available at https://catalogue.data.wa.gov.au/dataset/ (Title: DBCA – Legislated Lands and Waters [DBCA‐011]). Biologically Important Area for foraging by flatback turtles (© Commonwealth of Australia, Department of Climate Change, Energy, the Environment and Water, 2021) shapefile data available at http://www.environment.gov.au/fed/ (Title: Biologically Important Areas of Regionally Significant Marine Species). Kimberley Port Authority (© Western Australian Land Information Authority, 2023) shapefile data used and reproduced by permission from Western Australian Land Information Authority, Landgate, available at https://catalogue.data.wa.gov.au/dataset/ (Title: Port Authorities [LGATE‐243]). Pearl farm aquaculture leases boundary (© State of Western Australia, Department of Primary Industries and Regional Development, 2024) shapefile data available at https://catalogue.data.wa.gov.au/dataset/ (Title: Aquaculture Sites [DPIRD‐001]).
