# Supplementary material for: Incorporating fine‐scale behaviors into habitat suitability modeling: A case study for sea turtles
Source: Ecol Appl. 2025 Sep 12;35(6):e70095. doi: 10.1002/eap.70095 (PMC12426729; doi:10.1002/eap.70095)
Supplement: Supplementary file 1 — Appendix S1. [file EAP-35-e70095-s001.pdf]

APPENDIX S1

Journal: Ecological Applications

TITLE: Incorporating fine-scale behaviors into habitat suitability modeling: A case study for sea turtles

Authors: Jenna L Hounslow, Sabrina Fossette, Arnold van Rooijen, Anton D. Tucker, Scott D. Whiting, Adrian C. Gleiss

APPENDIX S1

Section S1: Additional methods

Delft3D-FLOW hydrodynamic model

Hourly tide-driven water levels and current velocity (flow components along east and north) were simulated over a representative 2-week spring-neap tidal cycle (1 – 15 February 2020, excluding model spin-up time) using Delft3D-FLOW (version 4.04.01; Lesser et al. 2004). The model was set up using Delft Dashboard (Van Ormondt et al. 2020), and extended from 121.9° to 122.5° longitude and -18.36 to 17.84° latitude with a grid resolution of 100 m. The model bathymetry was based on the same bathymetry data used in the HSMs; a high-resolution digital elevation model (DEM) of the North West Shelf with 30 m resolution (Lebrec 2021). Offshore water level boundary conditions were derived based on the global TOPEX/Poseidon dataset (TPXO 8.0; Egbert & Erofeeva 2002). All other model settings were kept at default values. Model output was validated by comparing simulated to observed tidal constituents and hourly water levels recorded at the Broome tide gauge (Station number 003102; Australian Government Bureau of Meteorology 2022) (Figure S1).

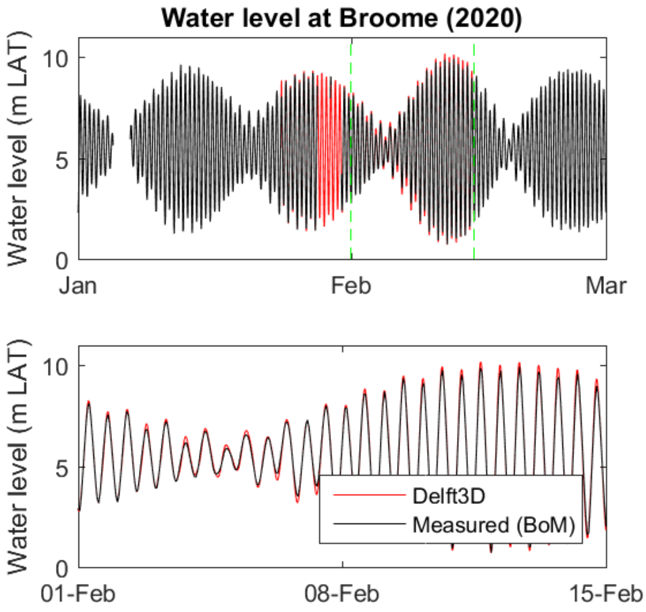

Figure S1 The Delft3D-Flow hydrodynamic model was validated by comparing simulated water level data (red line) to measured water level data (black line; observations from Broome tide gauge for 1-15 February 2020), Australian Government Bureau of Meteorology, 2024

APPENDIX S1

Table S1 Summary of adult flatback turtles (n = 42) captured and equipped with a multi-sensor biologging tag (CATS-Cam or CATS-Diary) at Yawuru Nagulagun Roebuck Bay in Western Australia, and geolocated behavior-labeled dives from (Hounslow et al. 2023), used as behavior-specific presence data for habitat suitability models.

ID	Sex	CCL (cm)	Mass (kg)	Deployment Date & Time (AWST)	Geolocated dives (*n*)	
					Forage	Rest
**6**	M	90.6	83.5	27/08/2018 15:56:45	46	45
**7**	M	85.5	69.5	28/08/2018 16:45:35	84	27
**8**	F	87.2	84.5	27/08/2018 18:17:34	71	93
**9**	M	86.8	70.5	28/08/2018 18:19:00	104	92
**10**	F	87.4	84.5	28/08/2018 17:26:00	108	41
**11**	M	81.2	62.0	24/06/2019 18:05:23	22	16
**13**	F	84.0	84.0	24/06/2019 18:23:13	25	14
**15**	M	89.9	82.5	27/06/2019 13:17:00	17	21
**17**	F	89.0	81.0	25/06/2019 15:49:35	6	4
**18**	F	89.1	77.0	21/08/2019 17:44:52	8	5
**19**	F	89.3	83.0	23/08/2019 13:14:59	2	8
**20**	F	86.3	68.0	23/08/2019 17:10:00	74	62
**22**	M	82.3	60.0	23/08/2019 18:18:24	92	86
**23**	F	86.0	67.8	19/02/2020 12:52:08	22	20
**24**	M	84.9	-	19/02/2020 14:33:48	6	38
**25**	F	-	80.0	17/02/2020 16:01:38	2	8
**26**	F	85.8	-	19/02/2020 13:42:25	9	13
**27**	F	72.5	46.0	19/02/2020 11:47:14	161	121
**28**	F	84.0	75.0	21/02/2020 1:47:39	267	109
**29**	F	91.2	82.5	20/02/2020 18:20:32	232	84
**30**	F	90.3	77.0	20/02/2020 11:32:30	268	76
**31**	M	86.6	77.0	20/02/2020 13:30:09	106	128
**32**	M	84.4	66.0	21/02/2020 13:29:00	0	11
**33**	F	98.9	97.0	21/02/2020 14:02:46	5	4
**35**	F	94.3	90.0	11/08/2020 13:25:24	25	14
**36**	F	87.3	74.0	11/08/2020 13:52:22	13	11
**37**	F	93.8	75.0	11/08/2020 15:58:00	123	107
**39**	F	86.6	75.0	11/08/2020 17:54:15	142	127
**40**	F	85.1	75.0	11/08/2020 18:34:30	62	44
**41**	F	92.1	86.0	12/08/2020 12:26:00	56	35
**42**	F	87.1	70.0	12/08/2020 15:58:00	35	44
**43**	F	87.3	80.0	13/08/2020 13:05:30	79	73
**44**	M	84.9	62.0	13/08/2020 15:32:44	34	41

APPENDIX S1

**45**	M	84.1	65.0	13/08/2020 17:03:00	3	0
**46**	M	86.4	67.0	14/08/2020 13:39:00	25	26
**47**	M	80.5	62.0	14/08/2020 15:31:00	29	15
**48**	M	88.9	75.0	18/08/2020 13:47:30	20	14
**49**	F	84.6	70.0	18/08/2020 14:38:55	120	157
**50**	F	90.2	91.0	18/08/2020 15:14:19	5	2
**51**	M	83.5	60.0	4/05/2021 15:54:16	135	88
**52**	M	87.9	65.0	4/05/2021 17:39:00	13	22
**54**	F	88.0	72.0	7/05/2021 14:11:00	2	2
**55**	M	86.5	67.0	7/05/2021 15:46:00	46	45
**56**	M	87.0	85.0	7/05/2021 17:26:00	84	27
				Total	***2658***	***1948***

CCL = curved carapace length, Mass estimated ± 1.5 kg

APPENDIX S1

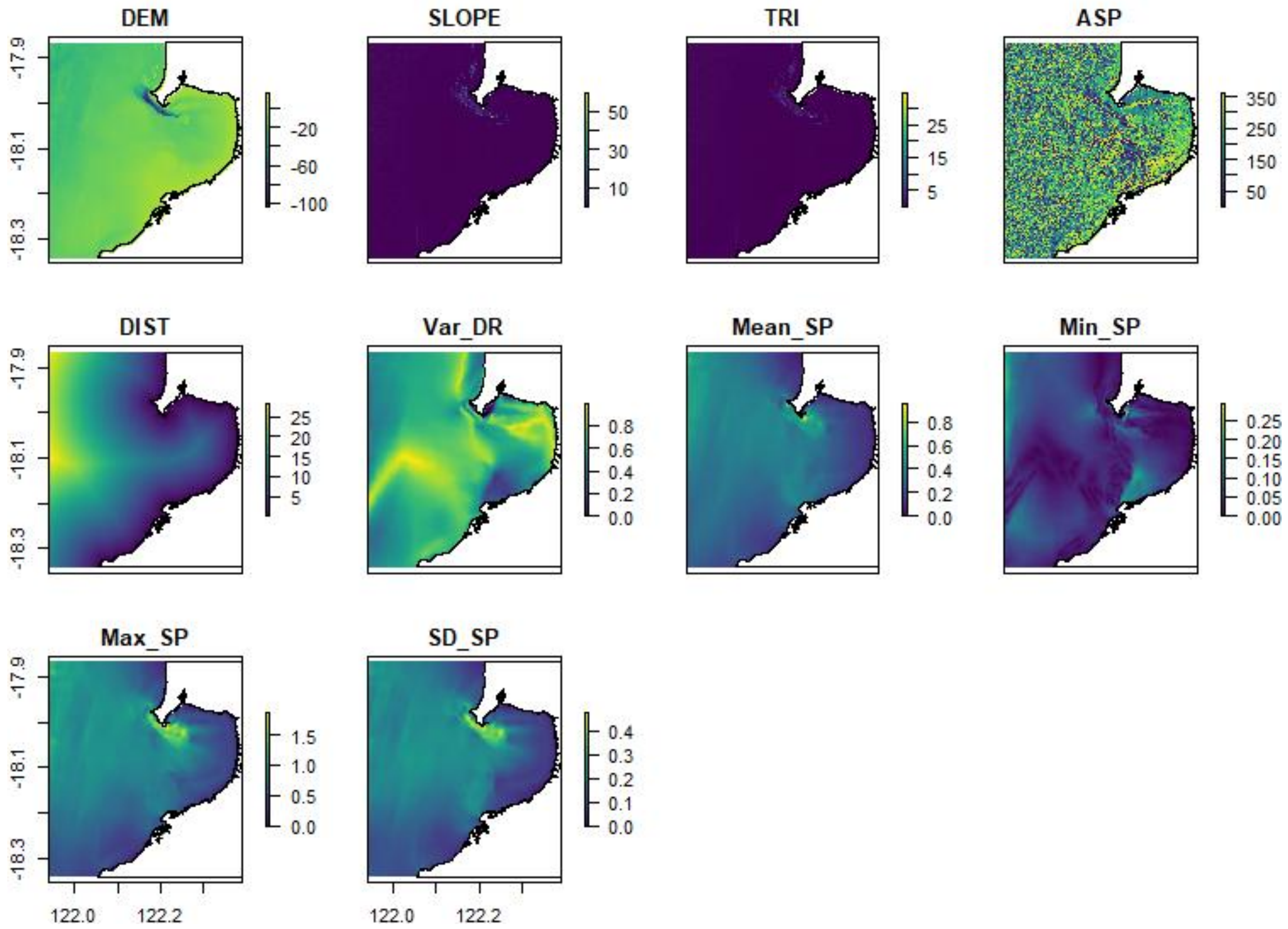

Figure S2 Environmental variables (*n* = 10) prepared for low water level (< 4 m) at Yawuru Nagulagun Roebuck Bay, Western Australia. For variable acronyms refer Table 2 in main text.

APPENDIX S1

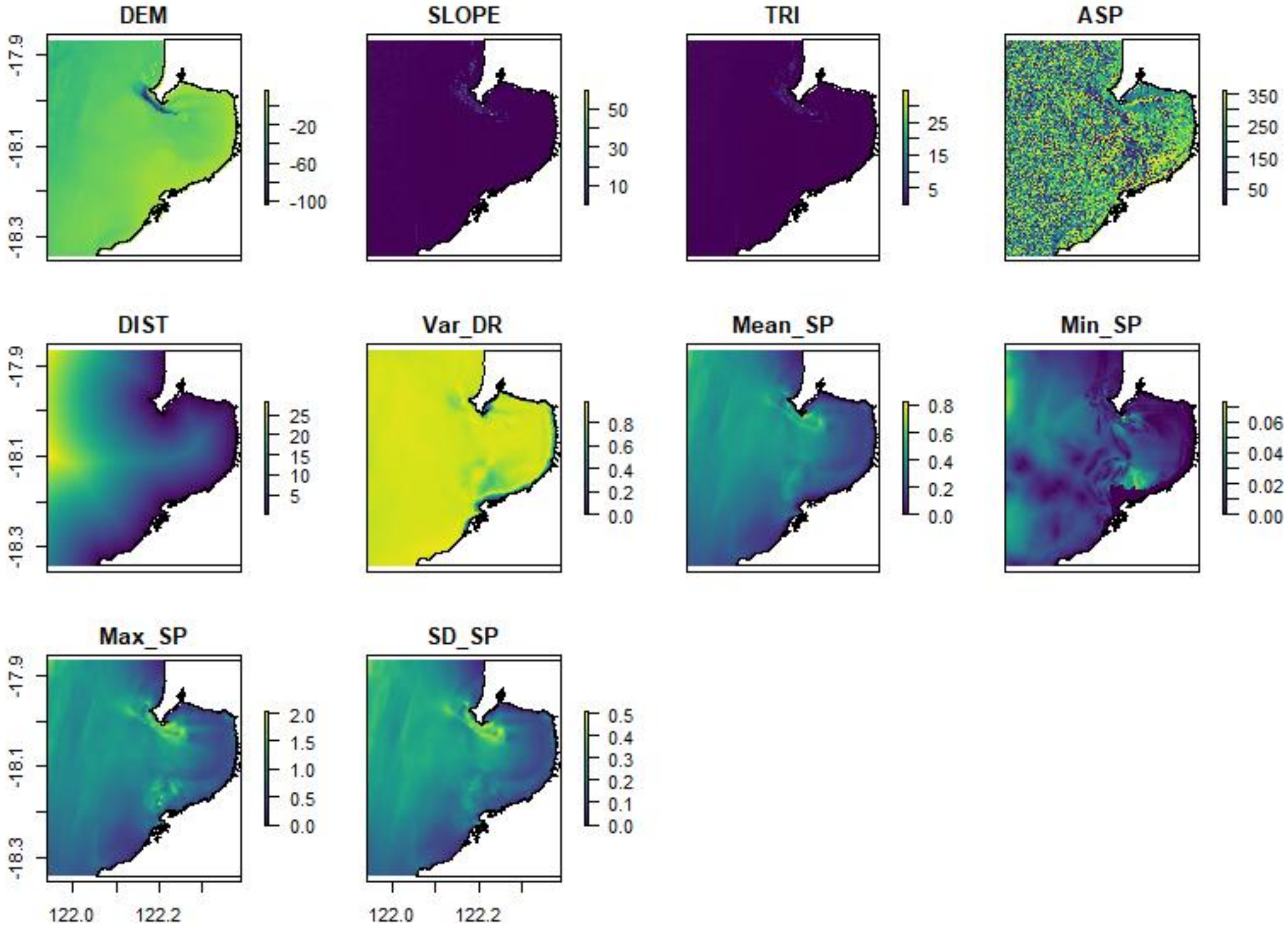

Figure S3 Environmental variables (*n* = 10) prepared for mid water level (4 -7 m) at Yawuru Nagulagun Roebuck Bay, Western Australia. For variable acronyms refer Table 2 in main text.

APPENDIX S1

.

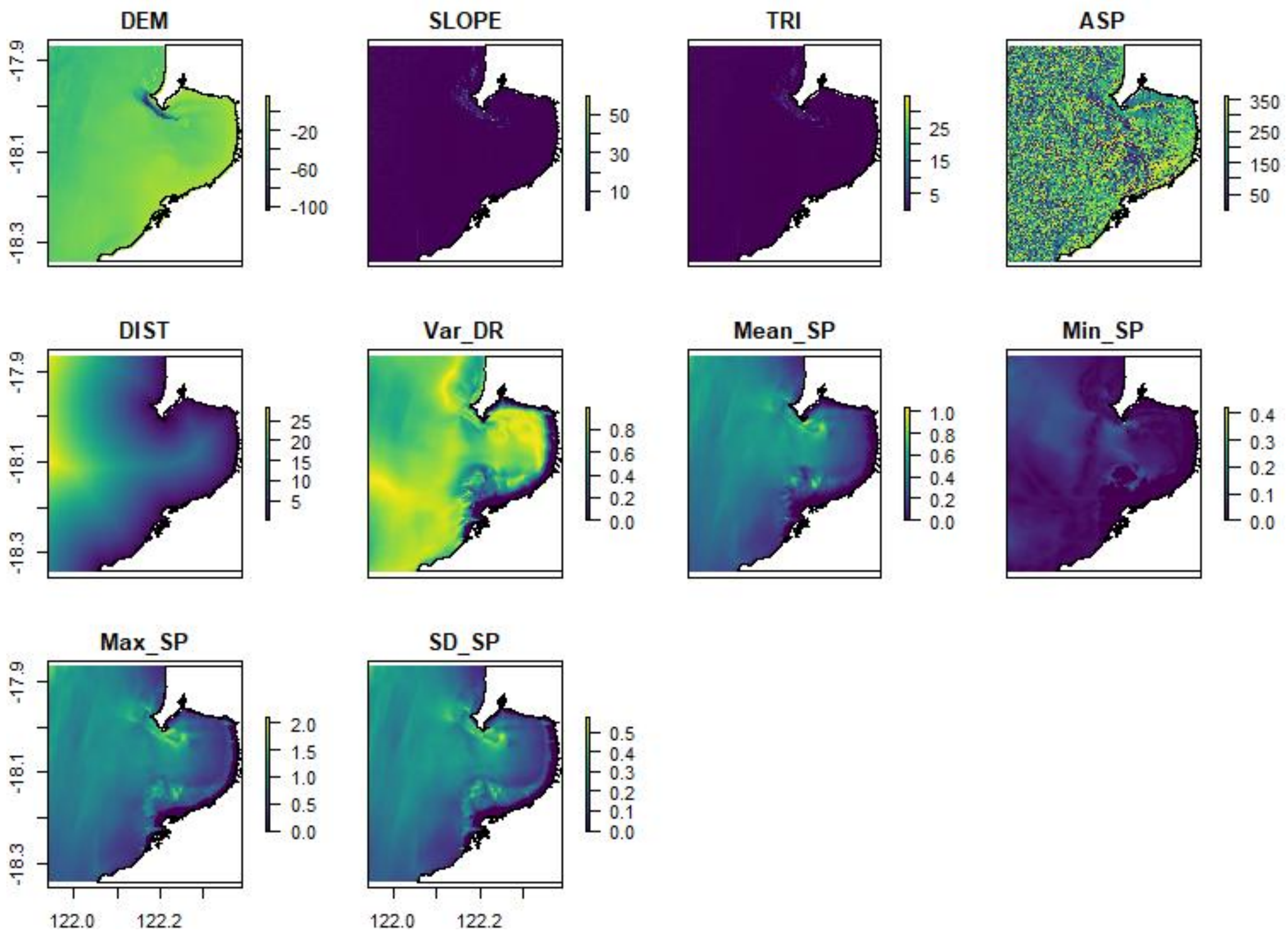

Figure S4 Environmental variables (n = 10) prepared for high water level (> 7 m) at Yawuru Nagulagun Roebuck Bay, Western Australia. For variable acronyms refer Table 2 in main text.

Behavior-specific presence-background data

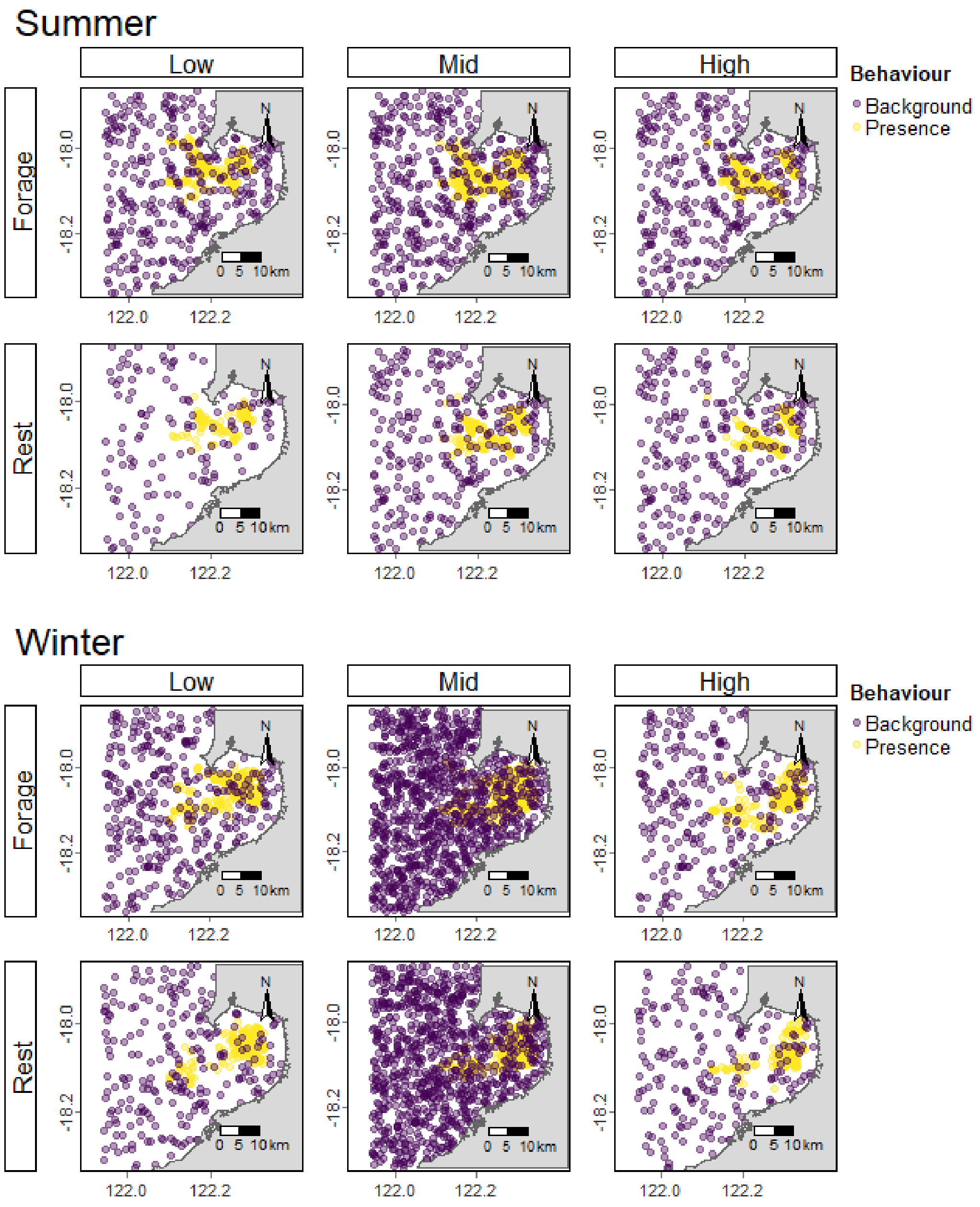

Figure S5 Map showing the locations of in-water foraging and resting presence-background data used for extracting environmental variable data as input to behavior-specific habitat suitability models (HSMs) for flatback turtles (*n* = 42) at Yawuru Nagulagun Roebuck Bay, Western Australia during summer (top) and winter (bottom). Panel row = behavior, panel column = water level category attributed to tide height. Behavioral presence data = yellow circles, background data = purple circles.

Section S2: Additional Results

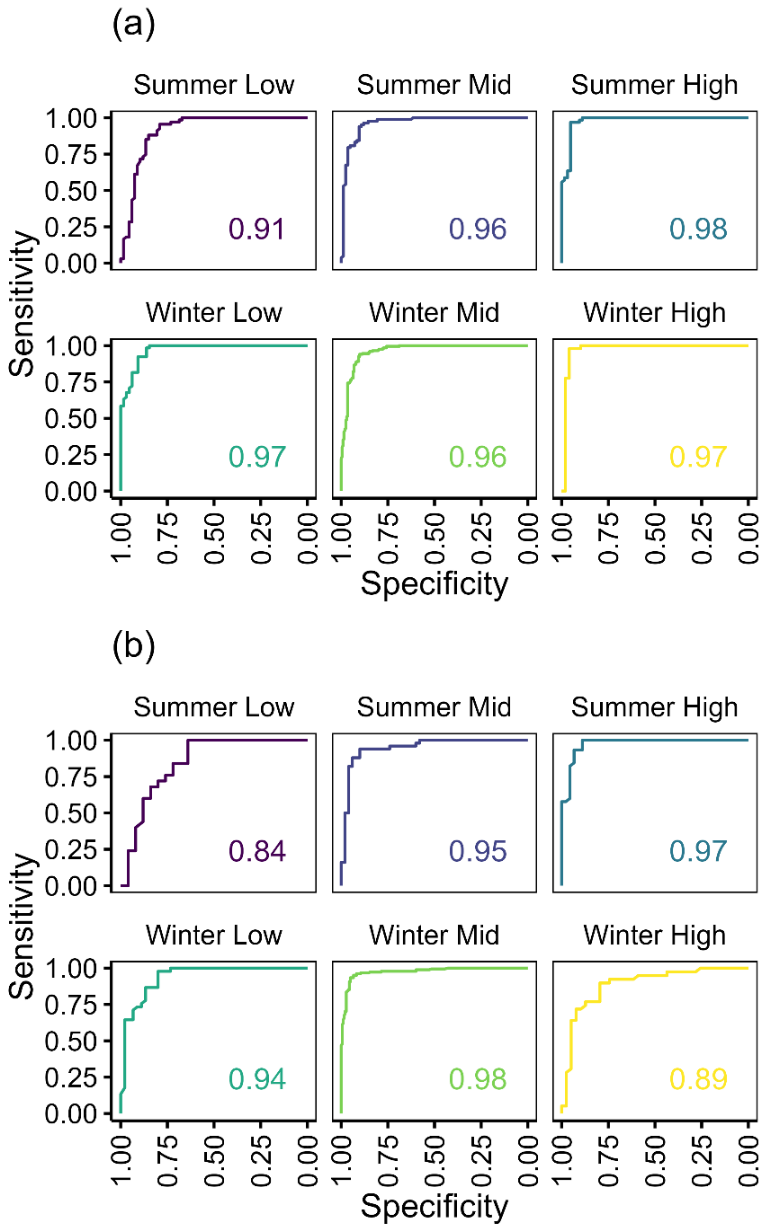

Figure S6 Receiver operating curves for Random Forest behavior-specific habitat suitability models for in-water (a) foraging and (b) resting by flatback turtles (*n* = 42) at Yawuru Nagulagun Roebuck Bay, Western Australia. Panel row = Season, panel column = water level category attributed to tide height, panel label = area under curve (AUC).

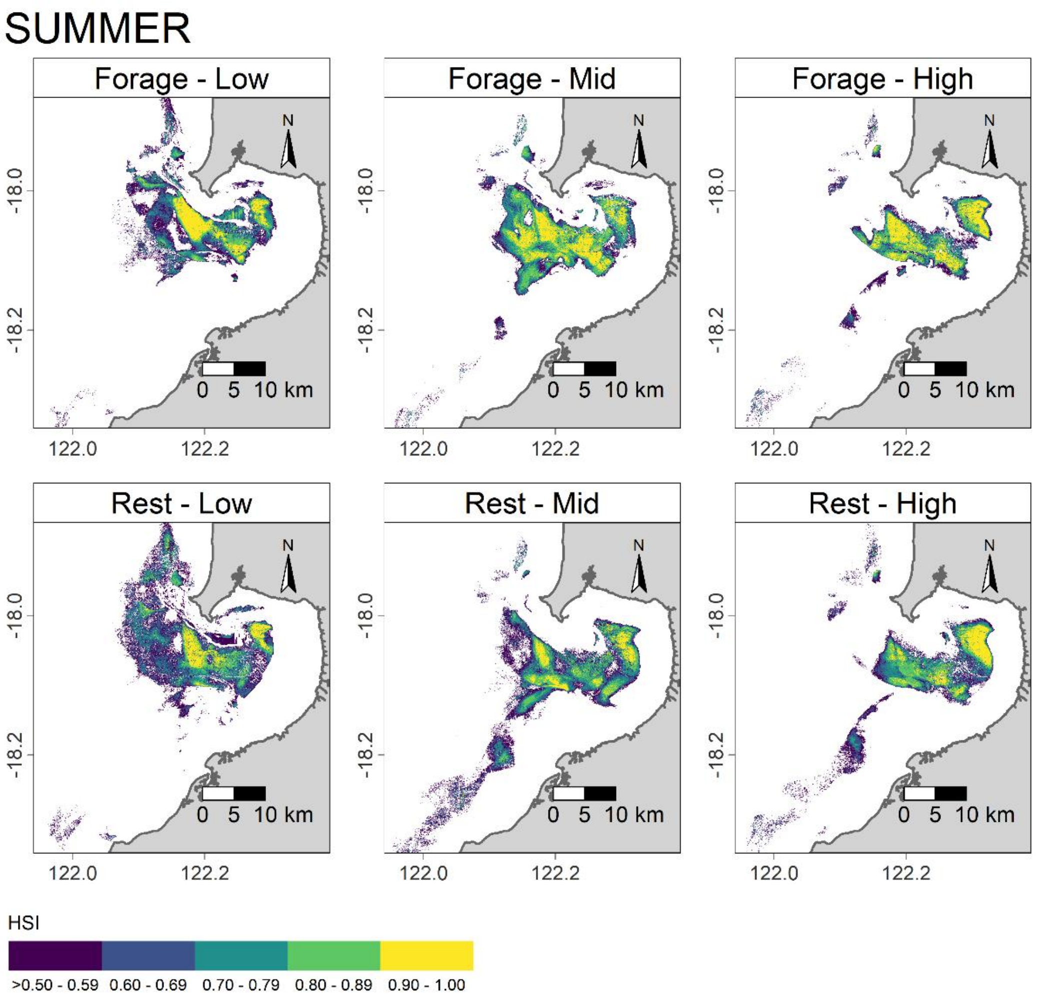

Figure S7 Behavior-specific habitat suitability for in-water foraging and resting by flatback turtles at Yawuru Nagulagun Roebuck Bay, Western Australia. Habitat Suitability Index (HSI) shows most suitable habitats (HSI > 0.5; unsuitable habitats coloured white) for foraging (top row) and resting (bottom row) during summer, according to water level categories attributed to tide height (Low < 4 m, Mid 4 – 7 m, and High > 7 m respectively).

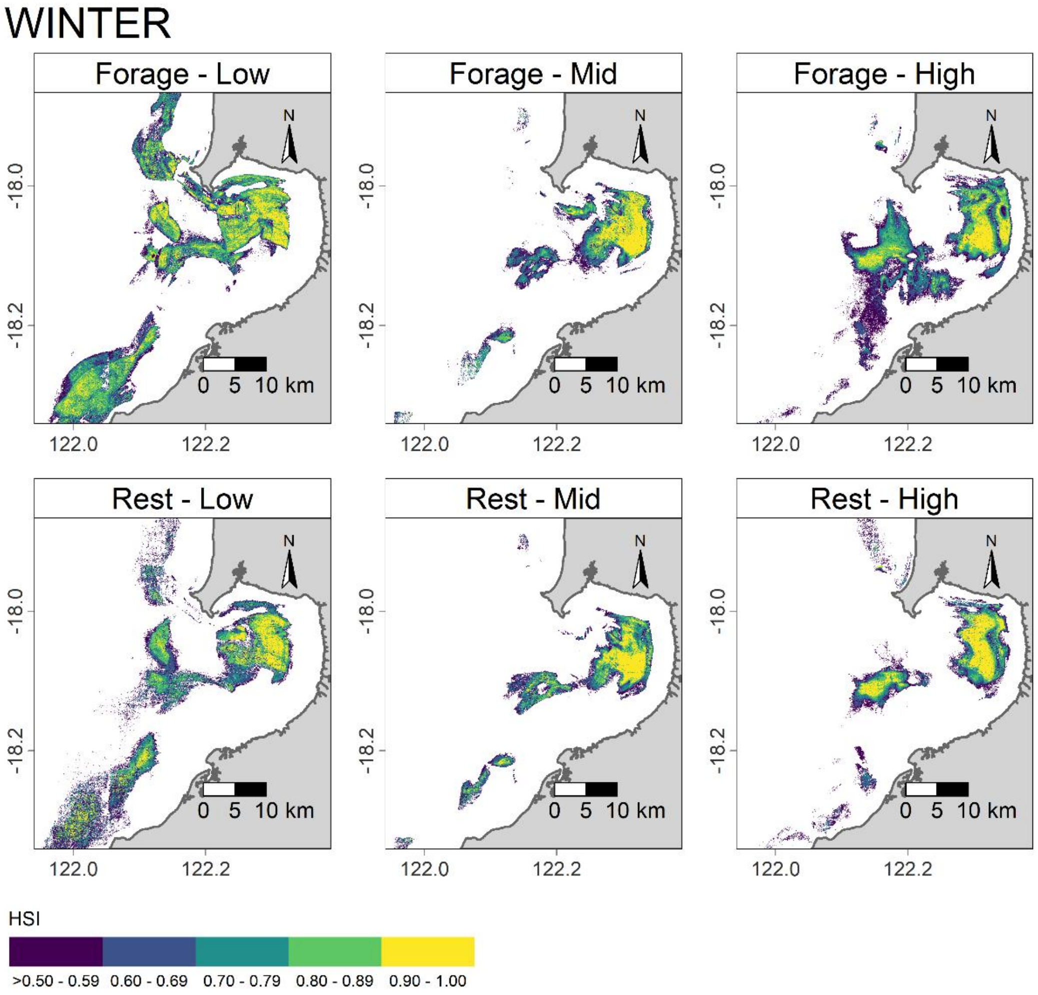

Figure S8 Behavior-specific habitat suitability for in-water foraging and resting by flatback turtles at Yawuru Nagulagun Roebuck Bay, Western Australia. Habitat Suitability Index (HSI) shows most suitable habitats (HSI > 0.5; unsuitable habitats coloured white) for foraging (top row) and resting (bottom row) during winter, according to water level categories attributed to tide height (Low < 4 m, Mid 4 – 7 m, and High > 7 m respectively).

References

Australian Government Bureau of Meteorology (2022) Australian Baseline Sea Level Monitoring Project Hourly Sea Level and Meteorological Data. Accessed 6 July 2022. http://www.bom.gov.au/oceanography/projects/abslmp/data/index.shtml

Egbert GD, Erofeeva SY (2002) Efficient inverse modeling of barotropic ocean tides. Journal of Atmospheric and Oceanic technology 19:183-204

Hounslow JL, Fossette S, Chong W, Bali R, Tucker AD, Whiting SD, Gleiss AC (2023) Behaviour-specific spatiotemporal patterns of habitat use by sea turtles revealed using biologging and supervised machine learning. Journal of Applied Ecology

Lebrec U (2021) High-resolution digital elevation model of the North West Shelf – 30m. Accessed December 17 2022. https://ecat.ga.gov.au/geonetwork/srv/eng/catalog.search#/metadata/144600

Lesser GR, Roelvink Jv, van Kester JTM, Stelling G (2004) Development and validation of a three-dimensional morphological model. Coastal Engineering 51:883-915

Van Ormondt M, Nederhoff K, van Dongeren A (2020) Delft Dashboard: a quick set-up tool for hydrodynamic models. Journal of Hydroinformatics 22:510-527
